# Assessing the Ecological Network of Svalbard Through Scaled Interaction Strength Data: Insights From a Century of Research

**DOI:** 10.1002/ece3.73318

**Published:** 2026-03-30

**Authors:** Mikhail K. Zhemchuzhnikov, Tomas Roslin, Stephen J. Coulson, Joël Bêty, Anna Traveset

**Affiliations:** ^1^ Mediterranean Institute for Advanced Studies (IMEDEA, UIB‐CSIC) Esporles Balearic Islands Spain; ^2^ Department of Ecology Swedish University of Agricultural Sciences Uppsala Sweden; ^3^ Ecosystems and Environment Research Programme (ECOENV), Faculty of Biological and Environmental Sciences University of Helsinki Finland; ^4^ Department of Arctic Biology University Centre in Svalbard Longyearbyen Norway; ^5^ Département de biologie, chimie et géographie, Centre d'études nordiques (CEN) Université du Québec à Rimouski Québec City Quebec Canada

**Keywords:** Arctic, energy flow, food web, functional dynamics, polar region, Svalbard

## Abstract

The Arctic is a hotspot of environmental change, as demonstrated by various monitoring programs and studies north of the Polar Circle. As these activities primarily focus on detecting shifts in biodiversity and phenology, the functional dynamics of the terrestrial ecological community of Arctic systems remain comparatively understudied. Current research coverage of actual species interactions exhibits considerable temporal and spatial heterogeneity. Nevertheless, there have been numerous attempts to synthesize existing knowledge into conceptual frameworks, including for well‐studied regions such as Svalbard. However, these schemes often do not incorporate the idea of interaction strength. In this work, we aim to integrate existing knowledge on interaction strengths into a conceptual model of the Svalbard Ecological Network. In doing so, we also highlight current knowledge gaps and the challenges of establishing a robust baseline of species interactions in the region. Such current challenges cannot be overcome without coordinated efforts among multiple research groups.

## Graphical abstract

In this work, we integrate existing knowledge on interaction strengths into a conceptual model of the Svalbard Ecological Network. We also highlight current knowledge gaps and challenges in establishing a robust baseline of species interactions in the region.

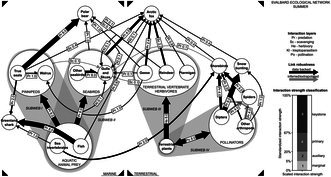

## 1 Ecosystem Monitoring in a Changing Arctic

One of the key aims of ecological monitoring is to describe changes in the state of species within communities over time. Importantly, communities consist not only of species but of the interactions between them. For this reason, communities can conveniently be described as networks, and our interest should focus on changes in the state of both nodes (species, or groups of species with a similar ecological role) and interactions (links) between them.

Nowhere are changes in ecological networks as topical as in the Arctic realm. This region is warming rapidly, with an overall pace of 0.73°C per decade, which is 3.8 times faster than the global trend (Rantanen et al. 2022). However, there is substantial spatial heterogeneity in this process, and some areas are heating even faster than expected from the general trend in the polar region (Taylor et al. 2022). Notably, Arctic areas span from regions exhibiting hotspots of biodiversity, for example, Taymyr (“Biodiversity in the Polar Regions in a warming world,” Meltofte 2018), to geographic edges providing a final northern limit for potential range expansions (e.g., Svalbard, as suggested by Jónsdóttir 2005).

To reveal the impact of ongoing climatic trends on Arctic communities and their phenology, long‐term terrestrial monitoring programs and systematic species inventories have been established in many Arctic locations (Berteaux et al. 2017; Christensen et al. 2020; Gillespie et al. 2020a, 2020b; Smith et al. 2020; Taylor et al. 2020). Many of these sites in the high Arctic zone cover decades of observations, including research stations on Bylot Island (Canada) (Gauthier, Berteaux, et al. 2024; Gauthier, Cadieux, et al. 2024), at Zackenberg (Greenland, BioBasis program) (Schmidt et al. 2023), and on the Svalbard archipelago (Norway, SESS) (Pedersen et al. 2022; Coulson et al. 2024). The programs pursued at these sites are mostly focused on trends in the diversity and abundance of nodes (often presented as species, other taxon or functional guilds) or resolving the interaction networks (metawebs) around particular nodes (e.g., modular approach in Climate‐ecological Observatory for Arctic Tundra (COAT) (Pedersen et al. 2022)). Nonetheless, even among these sites, monitoring methodology is generally not standardized and spatial coverage often remains limited (Thyrring et al. 2025) – with the notable exception of targeted initiatives, such as International Polar Years (Barr and Luedecke 2010), COAT (Pedersen et al. 2022), or smaller programs/projects targeting particular circumpolar species (Vongraven et al. 2012; Berteaux et al. 2017; Smith et al. 2020; McCabe et al. 2024; Blok et al. 2026). In terms of links (interactions between nodes), data often lack continuity, both in space and time, within and across years. This lack of standardization and spatial coverage complicates our understanding of functional changes in the Arctic biome. Although diversity and abundance trends can give insight into ecosystem state, they often cannot reveal the reasons for transitions from one state to another (Taylor et al. 2020; Saulnier‐Talbot et al. 2024). For most areas, there is no measured baseline for these interactions. Thus, our understanding of the complex puzzle of the Arctic ecological network remains constrained.

## 2 Current Knowledge about the Arctic Ecological Network

**FIGURE 1:**
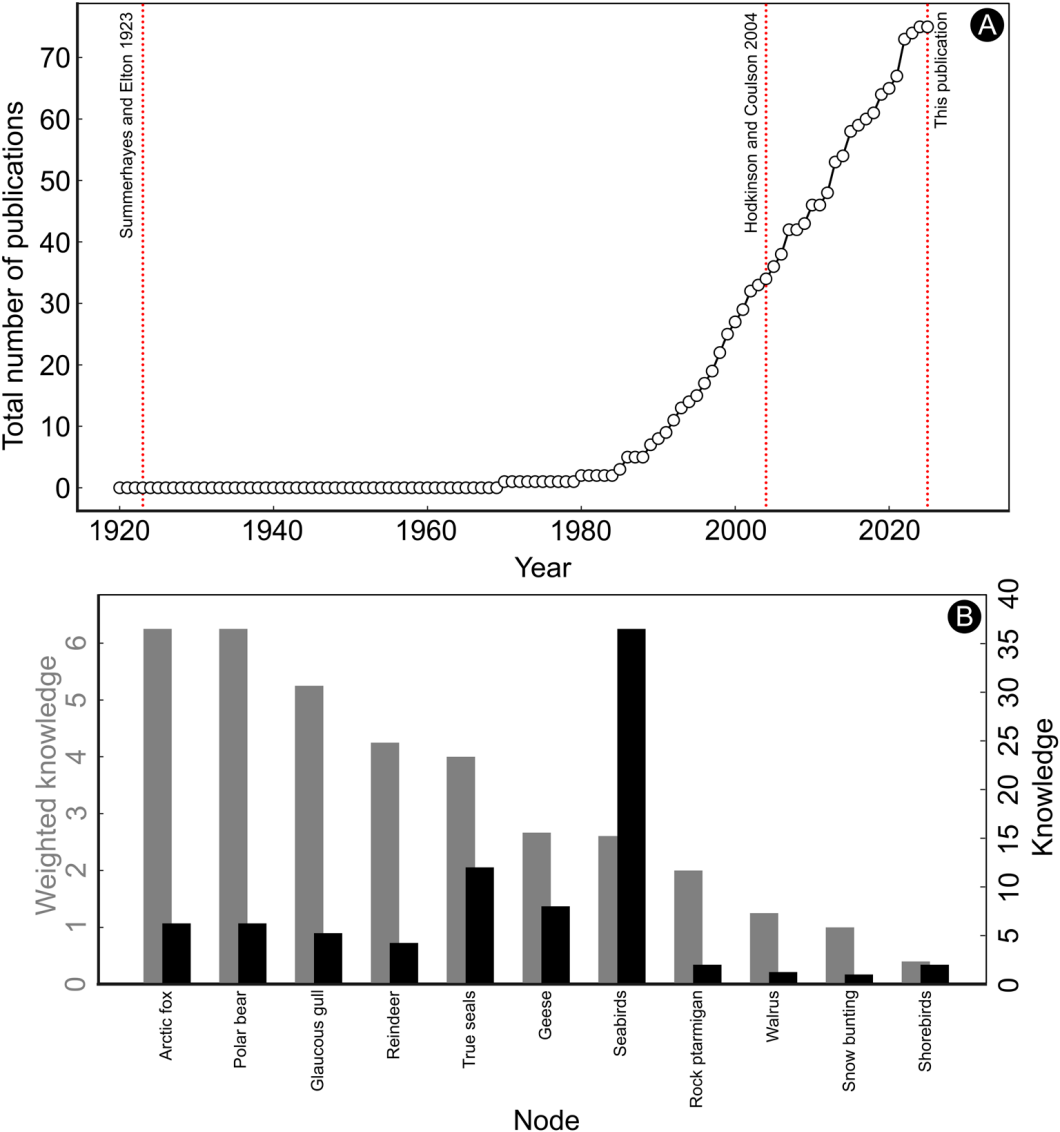
(A) The accumulation of literature on interactions in Svalbard over time, as here used for creating the weighted multilayered network (Appendix S4). (B) Weighted knowledge on nodes of above ground/sea level ecosystem in Svalbard, based on our literature review. Knowledge score is based on the number publication‐species pairs: To calculate this score, we gave a score of 1 for each species studied in a single paper. We gave the score of 0.25 instead of 1 if it was a case study (no interaction strength was measured). Afterwards, we summed the scores for the total. Weighted knowledge score was calculated as a ratio of knowledge about a node to the number of species in this node. For example, species from a certain node were studied in 3 papers: One of them was a study on interaction strength for species A (score = 1), another one—for species A and species B (score = 2), the last one was a case study for species C (score = 0.25). The sum of knowledge is 3.25. The weighted knowledge is corrected for the number of species in the node (3) = 3.25:3 = 1.08. The review of seabirds by Barrett et al. (2002) was not considered for calculating the scores. Glaucous gull was set aside of the other seabirds, as one of the most well‐studied bird species in the area regarding the interaction strength aspect. Seabirds include the most common regular breeders (excluding Glaucous gull).

The species richness (node diversity) of Arctic networks is relatively low when compared to other regions. Still, even in the Arctic, research efforts directed at studying various life forms are unbalanced. The main focus is usually on “hot topics”, so‐called “flagship species” (Rode et al. 2024), which are visually attractive, media‐popular species receiving high public and governmental attention. Typical polar flagship species are the polar bear *Ursus maritimus* , the Arctic fox *Vulpes lagopus* , reindeer *Rangifer tarandus* , geese, pinnipeds, and whales. Less often, they are shorebirds and seabirds (summarized for Svalbard in Figure 1). Although not applicable to rodent‐free Svalbard, multi‐annual cycles in small Arctic mammals (lemmings) and their effects on trophic networks have been extensively studied in the Arctic (Bêty et al. 2002; Gauthier, Ehrich, et al. 2024; Krebs 2024; Schmidt et al. 2012; Zhemchuzhnikov et al. 2024). Yet, most of the biomass in the Arctic and the majority of interactions involve invertebrates, primary producers, and fungi (Legagneux et al. 2014). Nonetheless, their roles in the network will often remain implicit and studied through their consumers (Stolz et al. 2023; Chagnon‐Lafortune et al. 2024). That often leads to a scenario in which ecological links are well resolved only around “flagship species”, and the overall network remains incomplete because of the directed research effort (De Aguiar et al. 2019). Implicitly, we are then assuming that interactions within the limited modules resolved are stronger than interactions beyond them, and that the links addressed will suffice to explain and predict system change. The resulting “grey area” of knowledge restricts us from creating an integrated picture of how the Arctic Ecological Network is organized. Indeed, the types of interactions in the Arctic biome are numerous (Krebs et al. 2003; Hodkinson and Coulson 2004; Descamps et al. 2017; Gauthier, Berteaux, et al. 2024). Thus, studying even a single type, or part of it, is a challenging task. An even more ambitious challenge is to correctly implement each interaction type into the overall configuration of an Arctic Ecological Network.

Current limit to building an ecological network restricts our understanding of ecosystem functioning, obscuring where the system's vulnerabilities lie and what impacts may arise from ongoing climatic changes. That is especially important for the most fragile ecosystems—islands, which are biodiversity reservoirs—and even more so for Arctic islands. Nonetheless, Arctic islands face the fastest rates of climate change, particularly in the Barents Sea and Kara Sea region (Rantanen et al. 2022). Here, the Svalbard archipelago and its waters provide a flagship case. This archipelago harbors large populations of polar bears, Arctic foxes, and reindeer, six pinniped species, three cetacean species, 45 regularly breeding bird species, and over one thousand freshwater and terrestrial invertebrate species (Hamilton et al. 2021; Coulson et al. 2024; “Checklist bird species in Svalbard,” 2024). At the same time, the Svalbard environment is heating rapidly, even when compared to other parts of the Arctic (You et al. 2021; Bradley et al. 2025). Such rates of change imply that local biodiversity is likely to be altered in the near future.

## 3 The Food Web of Bear Island as an Illustrative Case

Studies of Arctic food webs essentially started from Bear Island (Bjørnøya). This isolated island is part of the Svalbard archipelago, located at its southern range. Over a century ago, Summerhayes and Elton (1923) first outlined the food web of this island, in the form of a “nitrogen cycle.” This contribution painted the picture of a very simple web, with a total of 47 nodes. Nonetheless, this seminal web was based on a combination of different elements including species, groups of species, organic and inorganic matter, and nitrogen. Eighty years later, the interpretation was challenged by an updated scheme of the actual energy flow (Hodkinson and Coulson 2004). This was achieved by resolving “functional species” into the true component nodes consisting of tens of actual taxa with widely differing ecology. This new resolution uncovered the complexity of the arthropod web. The conceptual scheme of Hodkinson and Coulson (2004) was divided into a terrestrial invertebrate and vertebrate food webs, consisting of 31 and 7 nodes, respectively, as tied into a diverse interaction web. Indeed, when resolved at the species level, Arctic webs consist of a plethora of interactions hidden from the naked eye, but acting as the glue that holds the network together (Wirta et al. 2015; Schmidt et al. 2017).

While the paper by Hodkinson and Coulson is instructive, it dates back to more than 20 years ago and was not intended to be a detailed system description. With the implementation of new state‐of‐the‐art methods, such as DNA‐metabarcoding (Wirta et al. 2015), advances in our general knowledge of Svalbard species communities, and the development of new analytical tools (Pilosof et al. 2017), it is timely to update and upscale the scheme to the entire archipelago. While the Arctic is heterogeneous, and building a unified Arctic metaweb seems like a challenging goal, work in Greenland and Bylot Island has shown how this may be achieved for individual sites. For instance, for a species pool similar to that of Svalbard, Wirta et al. (2015) resolved a network fundamentally different in nature from the simple network suggested by Summerhayes and Elton (1923). This network also differed from the contribution of Hodkinson and Coulson (2004) in providing quantitative data. The same kind of quantification can now be achieved for the Svalbard network by integrating current knowledge about the strength of interactions between organisms.

**FIGURE 2:**
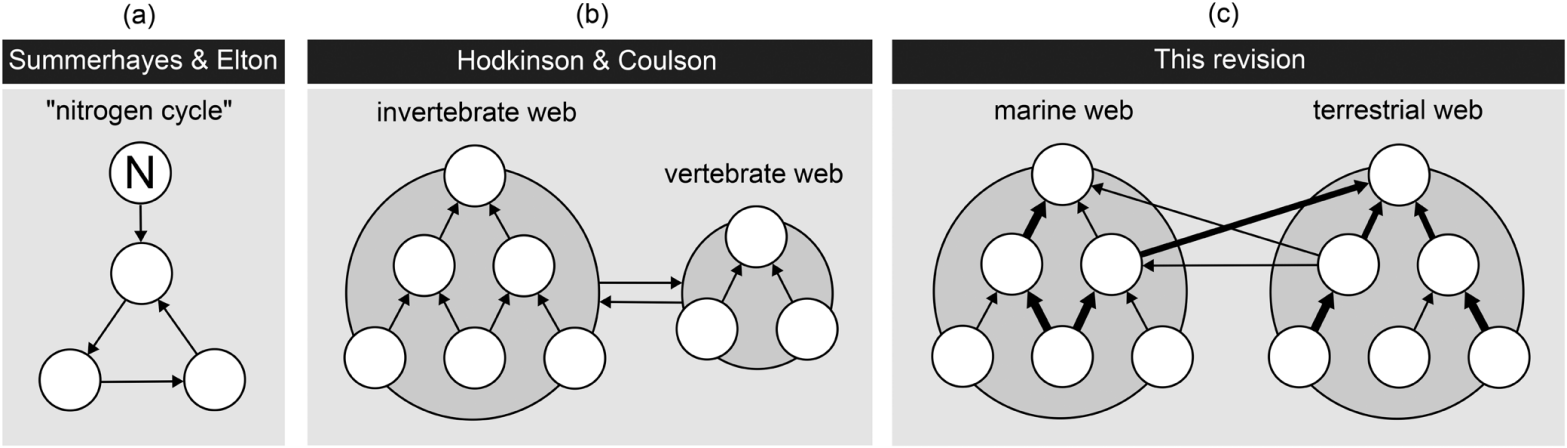
Conceptual illustration of consecutive revisions of the Svalbard ecological network architecture: (a) Summerhayes and Elton (1923), (b) Hodkinson and Coulson (2004), and (c) the present revision. The layout was simplified for clarity.

Here, we aim to revisit the original contributions of Summerhayes and Elton (1923) as revised by Hodkinson and Coulson (2004) (Table 1, Figure 2) to thereby (1) provide a historical perspective on studying ecological interactions on Svalbard; (2) reassess the model of Hodkinson and Coulson (2004) by creating a prototype of the multilayered ecological metaweb for Svalbard; and (3) reveal remaining information gaps by comparing Svalbard with other Arctic sites (Bylot Island and Greenland), along with proposed remedies.

**TABLE 1:** Comparison of conceptual schemes on the structure of the Arctic ecological network in Svalbard.

Scheme	Summerhayes and Elton	Hodkinson and Coulson	This revision
Journal, vol.	Journal of Ecology 11	Oikos 106	Ecology & Evolution
Year	1924	2004	2026
Geographical coverage	Local, Bjørnøya (Bear island)	Local, Ny‐Ålesund area	Regional, Svalbard
Focus	“Nitrogen cycle”	Invertebrate network complexity	(1) Overall network complexity (2) Interaction strength variation (3) Marine–terrestrial crossover links
Interaction strength	Not included	Not included	Included
Structure	No “elaborate chains” or “short‐circuiting” of the nitrogen cycle suggested.	Variety of invertebrate guilds linked by the plethora of interactions. Vertebrate web is very simple and has no elaborate chains	When weighted by the interaction strength, the ecological network presents a complex arrangement of links. They form energy flow “highways” and “country roads”
Schematic illustration	Figure 2a	Figure 2b	Figure 2c

## 4 Literature Survey

We used a structured algorithm for the literature survey (described in detail in the Appendix S5) to select papers on interactions in Svalbard and in the Barents' Sea region involving above‐ground and above‐sea‐level terrestrial and marine vertebrates and invertebrates. Briefly, we looked for papers in which (1) interaction strength, that is, the relative importance of a given interaction among all possible interactions, between consumer and resource was measured, or (2) a new, previously undescribed interaction was reported.

On the 5th of May 2025 (Scopus database), this yielded 704 and 239 papers across vertebrate and invertebrate surveys, respectively. Of these, only 11.4% and 0.4% contained data on actual interaction strength or novel interactions, respectively, which we found useful for updating the scheme of Hodkinson and Coulson (2004) (Figure 1A). The studies of “vertebrate” nodes had an obvious bias towards the “flagship species” (Figure 1B, Appendices S3 and S5, Table S1). This is not unexpected given the higher number of publications on resident species such as the Arctic fox and polar bear. In terms of invertebrates, only one study included Arctic pollinators (Gillespie and Cooper 2022), whereas another addressed arthropods as a food source for snow buntings (Stolz et al. 2023). In general, when considering terrestrial ecosystems, the below‐ground interactions are quantified rather poorly in comparison with above‐ground interactions in Svalbard. That was another reason for restricting our analyses to an above‐ground/above sea‐level part of the Svalbard ecosystem.

### 4.1 Scaling the Interaction Strength

**FIGURE 3:**
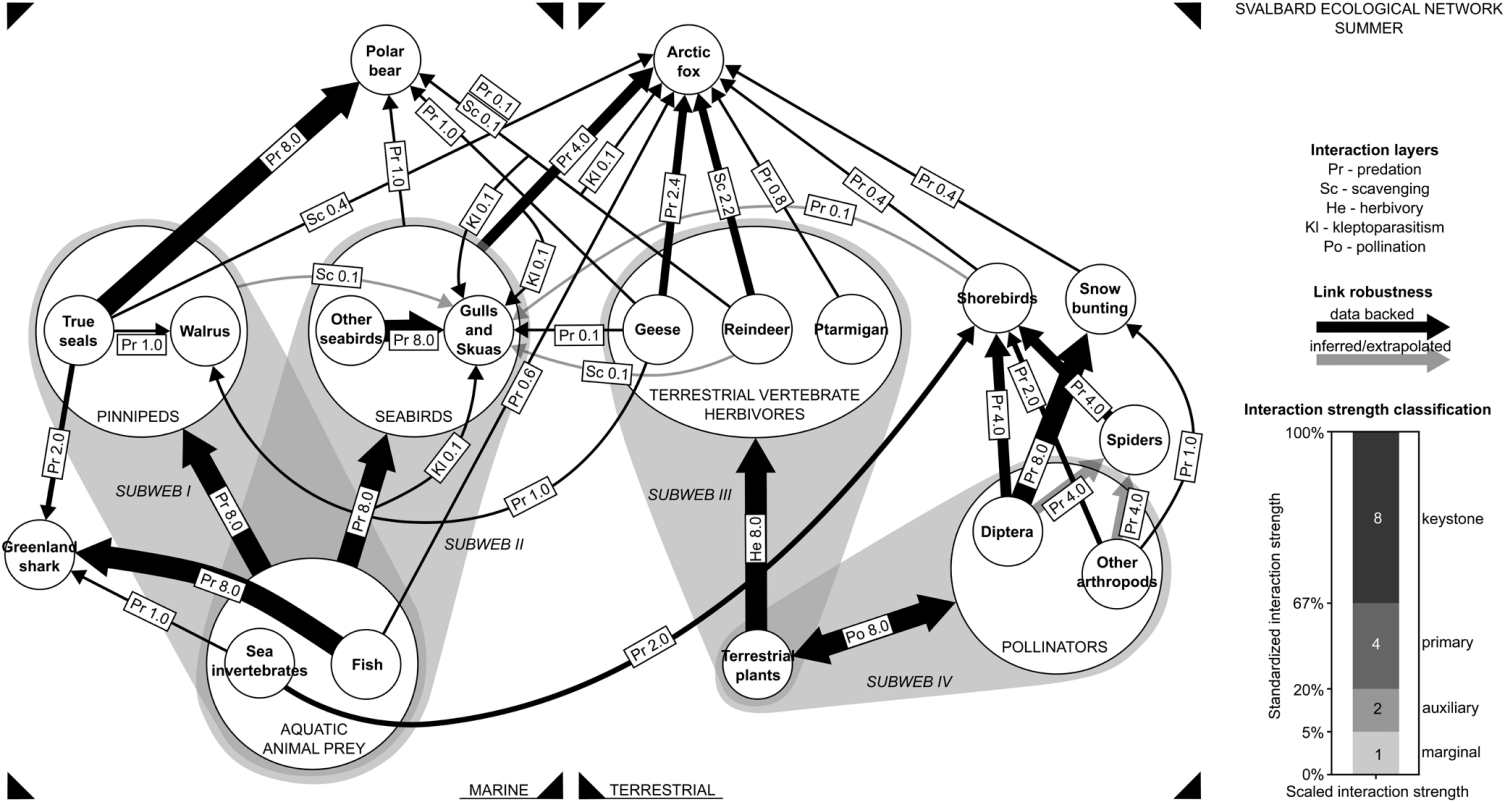
A partial metaweb of interactions occurring in the summer in the Svalbard archipelago, with a focus on terrestrial and marine mammals and birds, and terrestrial invertebrates. Different interaction layers are indicated with prefixes: He, herbivory; Kl, kleptoparasitism; Po, pollination; Pr, predation; Sc, scavenging. The number after the prefix indicates scaled interaction strength; the arrow width is proportional to that value. Transparent arrows indicate understudied or extrapolated interactions, which are highly likely to occur but have never been the focus of exploring the Arctic ecological network in Svalbard. Subwebs, the compartments of the current metaweb, are described in detail in Figure 4.

**FIGURE 4:**
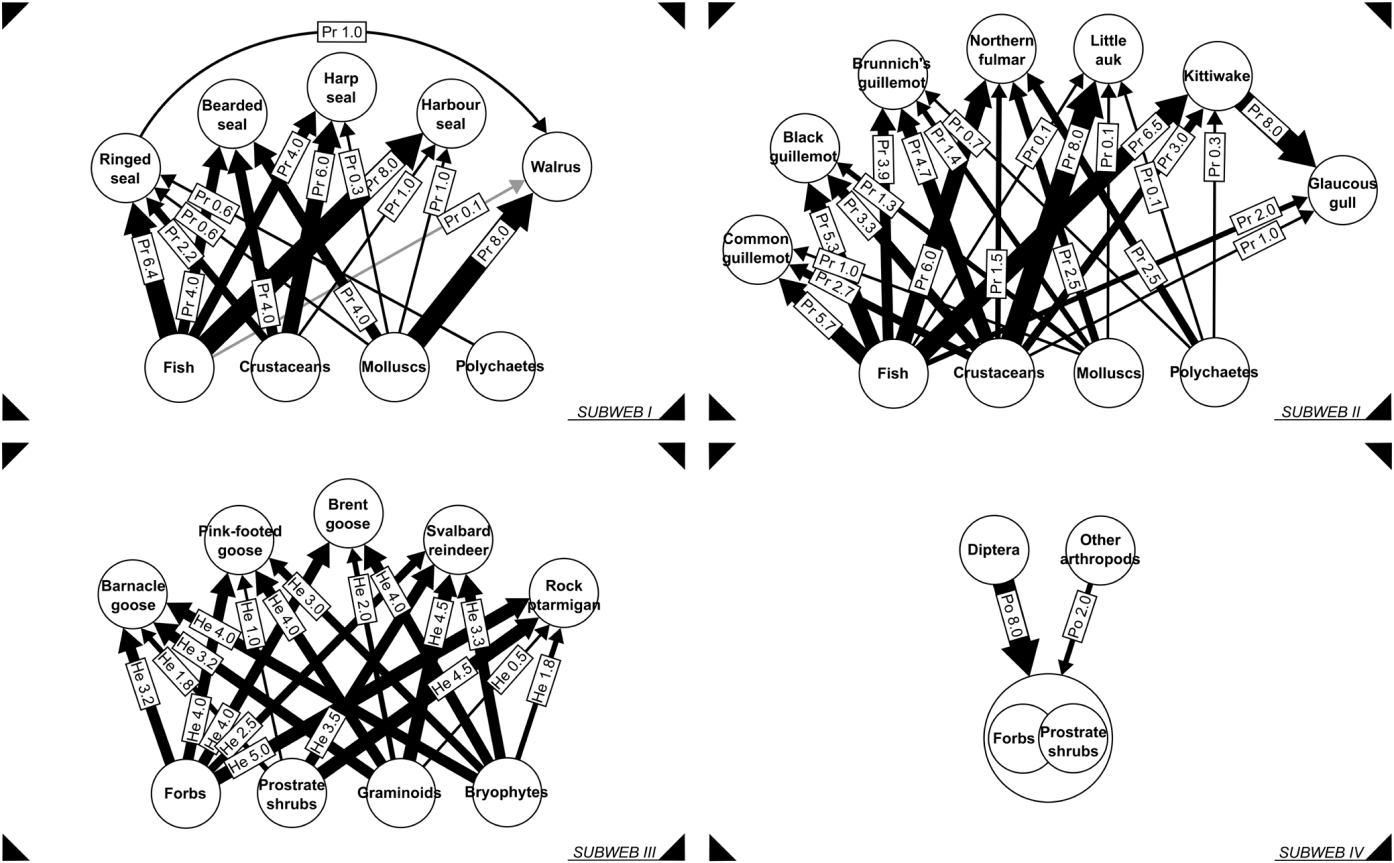
Subwebs of the metaweb in Figure 3. Subweb I—aquatic animal prey and pinnipeds, subweb II—aquatic animal prey and seabirds, subweb III—terrestrial plants and terrestrial herbivorous vertebrates, subweb IV—terrestrial plants and pollinators (phytocentric subweb). Only seabirds with the relevant data on their summer diet were included in subweb II. See Figure 3 caption for more details.

**FIGURE 5:**
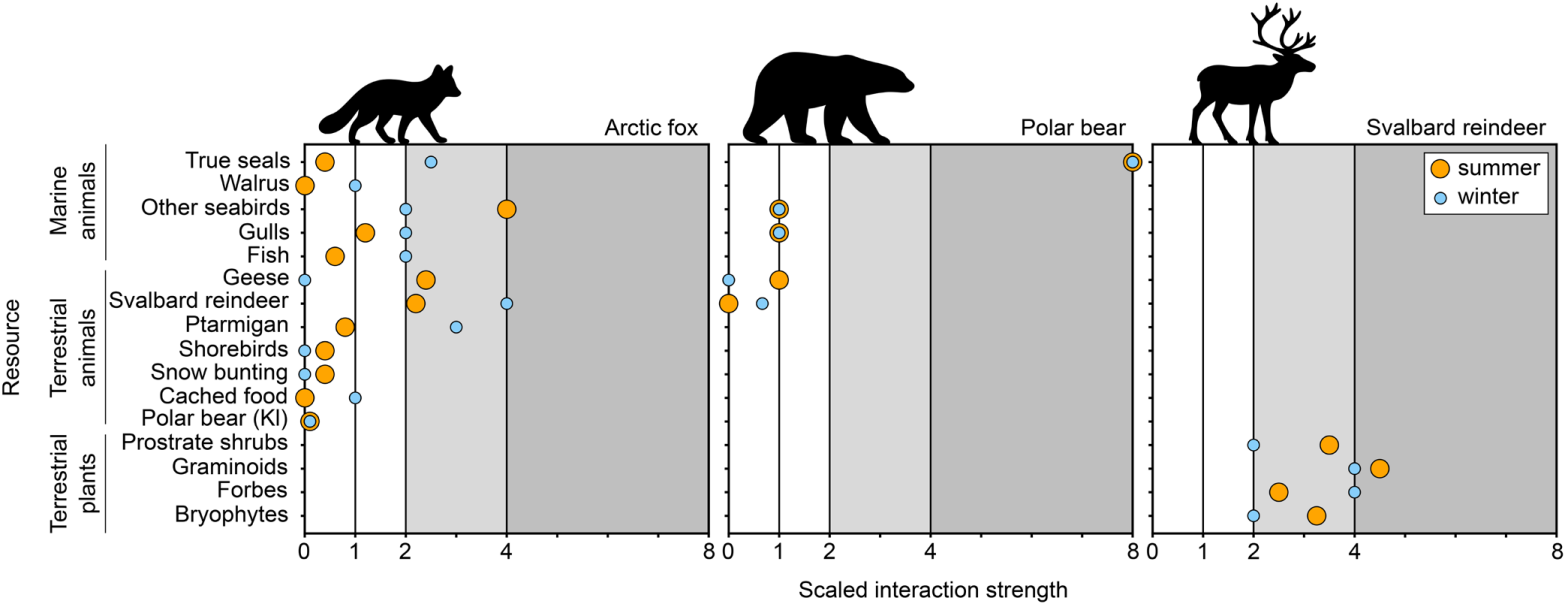
Scaled interaction strength between consumers (Arctic fox, Polar bear, and Svalbard reindeer) and their resources in Svalbard in summer and winter. Although vegetation is regularly found in the diet of Arctic fox and Polar bear, it is premature to make any robust inference about its ecological meaning, and whether it is consumed on purpose, as a secondary item, or unintentionally with the prey. Therefore, it is omitted from the plot. Polar bear (Kl) indicates kleptoparasitic use of polar bear prey.

We found that the studies recovered used diverse methods for recording and estimating the interaction strength between species, being neither consistent over different taxonomic units nor over time and space. Interaction strength included such metrics as the frequency of occurrence, the relative biomass consumed, relative caloric value, the whole‐scat equivalent, percentage of food fragments, relative importance index, and some other estimates (Appendix S1). To tackle this challenge, we (1) standardized the interaction value for each study‐consumer pair, so the sum of interactions was equal to 100%, (2) assigned interaction a score of 1, 2, 4, or 8 (with 0 indicating absence), following a log_2_ progression to reflect the nonlinear increase in relative interaction importance. Interactions were defined as follows: 0—*absence* of interaction (0%); 1—*marginal* interactions, which are weak, occasional interaction (anecdotal cases), not regular across years or within years (0%–5%); 2—*auxiliary* interaction, regular across years, but sporadic in space and time within year, with limited impact on consumers' fitness and survival, may be crucial when primary/keystone food source is not available (5%–20%); 4—*primary* interactions, which are crucial when the keystone resource is absent or scarce, or when the source is equally important with a few other resources (20%–67%); 8—*keystone* interactions, critical for the survival of the energy recipient (over 67%, when a given interaction is twice as strong as any other interaction) (Appendix S2). (3) As the last step, we averaged the scaled score for a consumer for winter and summer time periods, when several studies were available. Because scores were averaged across studies, intermediate values between the original scale categories could occur. These averaged values were retained and rounded to one decimal place (Figures 3, 4, 5, Appendix S5, Table S3). We classified each study as a “winter” or “summer” study based on whether the majority of the study period fell within winter months (November–March) or summer months (May–September). We also complemented the table with the case studies with an assigned minimal averaged score of 0.1. We extrapolated summer interaction strength towards winter interactions in several cases (Appendix S5, Table S3). Overall, the “winter” data were incomplete (Appendix S5, Figure S1); thus, we decided to focus only on a summer web in our inference, complemented by the data on a few species, for which winter and summer interaction strength estimates could be retrieved (Arctic fox, polar bear, and Svalbard reindeer).

The above methodology allowed us to build a semi‐quantitative directed unipartite network and to update and upscale the previous schematic one. Initially, we included the following interaction layers, where each layer depicted a certain ecological function: predation, scavenging, coprophagy, pollination, kleptoparasitism, herbivory, and detritus flow. Nevertheless, the data on coprophagy and detritus flow were too scarce to resolve the interaction layer well, and for that reason were not included in the metaweb. Based on the information gathered, we constructed a weighted multilayer ecological network for the Arctic, specifically for Svalbard, representing the summer season (Figures 3 and 4), marking the first such reconstruction for the entire Arctic region. In addition, we visualized the scaled strength of summer and winter interactions for top Arctic terrestrial and marine predators (Arctic fox, Polar bear) and for Svalbard reindeer (Figure 5).

In evaluating the current results, we stress that they are only as strong as the data underlying them. Biases in the focus invested in different taxa will reflect directly into the scores observed. We will return to these caveats later on.

## 5 Insights from the Multilayered Ecological Network of Svalbard

After the systematic survey of current literature on Svalbard interactions, focusing on above‐ground and above‐sea‐level vertebrate and invertebrate organisms, we present an updated version of the Svalbard ecological network conceptual scheme. This network collates recent knowledge on interactions in the area, and more importantly, is weighted by interaction strength. As with any generalized framework, it represents a metaweb that does not explicitly account for node co‐occurrence and, therefore, does not constitute a realized ecological network (Pecuchet et al. 2020; Parisy et al. 2025). It should instead be viewed as a higher‐level conceptual blueprint.

### 5.1 Apex Arctic Predators and Marine–Terrestrial Crossover

The previous schematics (Summerhayes and Elton 1923; Hodkinson and Coulson 2004) give a simplistic, unweighted overview of a terrestrial vertebrate web. Compared to this seminal entry, the new conceptual framework presents a visual and numerical demonstration of connectivity between marine and terrestrial Arctic biomes via coastal areas. The top marine and terrestrial predators in Svalbard, polar bear and Arctic fox, can utilize energy from both marine and inland ecosystems (Figures 3 and 5). To what extent they draw on each varies with the time of year and the availability of primary resources (Schmidt et al. 2022). It is important to note that the proposed interaction strengths reflect spatial and interannual averages. For instance, the Arctic fox summer diet varies with the fox's spatial distribution: if multiple resources are available in the area, foxes can feed on goose and seabird eggs and juveniles, as well as reindeer carcasses; however, individuals located inland rely on resources available near the den and therefore largely scavenge reindeer carcasses (Eide et al. 2005).

### 5.2 Seasonality in Network Structure

Although most of the data comes from the summer months, the summer‐ and winter‐time networks of Svalbard differ substantially in many aspects of network structure. The fair assumption would be that the summer‐time network is characterized by a greater number of nodes and links in comparison with its winter‐time configuration (Figure 3, Appendix S5, Figure S1). This difference is mainly due to the presence of migratory bird species in the summer, including seabirds, shorebirds, geese, and snow buntings, with rock ptarmigan being the only winter terrestrial resident, to the arthropod and plant community, and to the overall higher biomass. Such differences are typical of any environment with high seasonal differences in productivity, energy availability and availability to water (Ernakovich et al. 2014). Summer inflow of a high number of migratory species reshapes the structure of trophic webs, particular trophic cascades and the energy flow (Bauer and Hoye 2014). Moreover, nonbreeding areas, including both stop‐over sites and wintering grounds, act as convergence points, where migrants from different Arctic sites mix, interact, compete, and exchange genes and pathogens (Bauer and Hoye 2014; McDuie et al. 2024).

Unsurprisingly, summer and winter diets (and the degree to which predators rely on marine vs. terrestrial prey) also differ significantly for resident species (Lønø 1970; Prestrud 1992). The crossover flow in our conceptual model was largely due to the predation and scavenging activity of the top predators (Figures 3 and 5), the polar bear, and the Arctic fox, but also gulls and skuas. This crossover link may be lower in variety and weight during winter due to better accessibility of seals for polar bears, and increasing reliance on reindeer carcasses and probably cached food items by Arctic fox, given the absence of migratory birds. The interactions observed for top predators—particularly the Arctic fox (Figure 5)—reflect their broad involvement across multiple interaction layers, including predation, scavenging, and kleptoparasitism (Figures 3 and 5), spanning both the marine and terrestrial components of the system.

### 5.3 A Potential Key Role for Reindeer

The scheme of Hodkinson and Coulson (2004) implies that, in the rodent‐free tundra of Svalbard, the reindeer plays a key role in connecting nodes of terrestrial vertebrate web, and also links terrestrial vertebrate and invertebrate webs. When more nodes are added to the system and links are weighted, this remains true. The reindeer appears to be one of the key connection nodes of the terrestrial part of the ecosystem (Figure 3). As a primary local herbivore and a resource for top predators and scavengers (Figure 3, Figure S1), the reindeer bridges the food web. High biomass of primary producers supports stable populations of the reindeer, and its natural mortality supplies carcasses—which will in turn supply crucial food for inland Arctic foxes, which forage far from seabird or goose colonies during summer. The linkage that reindeer provide between marine and terrestrial ecosystems may also be important for polar bears during periods of ice retreat, when access to seals is limited. Polar bears have even been documented to hunt reindeer (Stempniewicz et al. 2021), and with increasing ice retreat, it may become a more common strategy. In summer, hematophagous arthropods may feed on reindeer blood, as in other Arctic sites (Koltz and Culler 2021). Moreover, reindeer carcasses may serve as an important organic source for plants and arthropods, though this role is currently understudied (Situnayake et al. 2025). In addition, reindeer have been shown to actively feed on geese droppings in Svalbard (Van Der Wal and Loonen 1998). Thus, the reindeer is involved in scavenging, predation, herbivory, coprophagy, parasitism and detritus flow, highlighting its key role in the Svalbard ecosystem. However, the latter three types of interactions remain largely overlooked in current research. The absence of reindeer (but also some other key nodes, e.g., geese species) on the isolated Bear Island (Summerhayes and Elton 1923) may explain an overall simpler configuration of the network there, compared to other parts of Svalbard.

## 6 Comparisons with Other Arctic Sites

In assessing the emerging view of the Svalbard metaweb, comparisons with the sites of Zackenberg (NE Greenland) and Bylot Island (Nunavut, Canada) were made. In brief, there were two reasons for choosing these sites. First, they represent model regions where substantial efforts have been invested in quantifying the links of the local ecological networks. As such, they may serve as templates for future efforts to be implemented in Svalbard. Second, while still in its infancy, the emerging quantification of the Svalbard network is still informative enough to allow for explicit comparisons between sites.

A major difference relates to the absence of rodents from Svalbard. There are no native rodent species in this region, only a few local introduced populations near some settlements (Fredga et al. 1990; Fauteux et al. 2021). This creates a profound difference to the situation on Bylot Island and in North‐East Greenland, where rodents play a key role in shaping the network. As a result, the nodal species of the terrestrial Svalbard network are different from that of Bylot Island or Zackenberg. At these sites, lemming species form a key prey for Arctic fox and avian predators. Importantly, the rodents' populations on Bylot Island and North‐Eastern Greenland follow multi‐annual cycles (Gauthier, Ehrich, et al. 2024), and the local interaction network shifts to alternative states in lemming‐rich and lemming‐poor years (Bergeron et al. 2026; Duchesne et al. 2021; Schmidt et al. 2022). Across the Arctic, predator–prey body size ratio may ultimately affect the type and frequency of “temporal switches” observed in the network. For example, large mammalian herbivores of the tundra can escape predation, be regulated by intra‐specific competition, and be often affected by stochastic climatic events. In contrast, small mammals are more often regulated by predation, and their population cycles may generate cascading effects in the entire network (Krebs 1996; Krebs et al. 2003).

A major knowledge gap in the Svalbard network suggested by studies conducted at Zackenberg is the fragmentary knowledge of the arthropod interaction layers. Despite high diversity across families and functional guilds, only a few studies have quantified interaction strengths (Gillespie and Cooper 2022; Stolz et al. 2023). At Zackenberg, consistent investment in resolving both the nodes and the links of arthropods has exposed their key role in pollination (Tiusanen et al. 2016), in predation on arthropods (Wirta et al. 2015), and as food for insectivorous birds (Wirta et al. 2015; Zhemchuzhnikov et al. 2025). Nonetheless, the Zackenberg fauna is markedly poorer (some 350 species; Wirta et al. 2016) than the Svalbard one (Coulson et al. 2025). That the arthropods of Svalbard may occupy an even more central role is suggested by the previous review from Bear Island (Hodkinson and Coulson 2004), which highlighted the extreme diversity of arthropod functional guilds and the diverse connections between the arthropods themselves, and between arthropods and other nodes of the terrestrial ecosystem. Thus, there is no doubt that our current representation of the Svalbard network remains biased until more effort is invested in resolving not only the species but their links and roles.

## 7 The Future of Arctic Ecological Interaction Networks

In this viewpoint, we have taken stock of the current knowledge of Arctic interactions in general and the Svalbard metaweb in particular. In doing so, we have uncovered three features:

First, even within a well‐studied site, the current state of knowledge on species interactions is fragmentary. Current representations are ridden by taxonomic and methodological biases. The taxonomic bias must be remedied by a conscious investment in all parts of the network. Just why this is needed is vividly illustrated by the changing perceptions of the Svalbard network from Summerhayes and Elton (1923) through Hodkinson and Coulson (2004) to the current contribution. Resolving the nodes at a more detailed taxonomic level and scaling the links between nodes will allow for a deeper insight into the architecture of the Arctic network. Such studies are needed to identify the main pathways of energy flow through the network.

Second, to advance our understanding of the functioning of the Svalbard ecosystem, as well as that of other Arctic areas, we must agree on some standardized approaches. Our literature survey has revealed a plethora of metrics collected by different techniques in different ways with little of a general denominator. Variation in methodology hampers general progress and insights. As a minimum requirement, we call for studies aiming to quantify node abundances and link strength at the species level, per explicit units of area. Without such quantitative data, there is no way to refine our current prototype of the quantified network. We urgently need such data to reliably quantify changes in the state of the ecosystem.

Third, the Arctic system is about to change. Causes include the potential spread of voles, outbreaks of new parasites or diseases in herbivores and predators, including altering the ecology of Influenza A Viruses and their hosts (Gass et al. 2022), new invasive species (Coulson 2015; Coulson et al. 2025), and northern range expansion of boreal species (Lai et al. 2022). In addition, earlier and more extensive retreat of the ice will force polar bears to rely on terrestrial foraging (Rode et al. 2015), and other aspects of climate change may shift the breeding patterns of birds (Lameris et al. 2021). An increase in the occurrence of rain‐on‐snow events may impact interactions in the terrestrial plant‐herbivore system, decreasing accessibility of food for reindeer (Rixen et al. 2022). There will be winners and losers. Some species will benefit while others may find no space, neither northward towards higher latitudes nor upward towards higher elevations, to track suitable conditions or prey phenology. If we want to be able to predict which changes are more likely to happen and what consequences they will bring, there is a pressing need to study hidden and indirect interactions (Roslin et al. 2013), standardize observation tools, and connect different interaction layers. Long‐term monitoring is particularly needed to determine which changes are part of natural rhythms or cycles, which fluctuations reflect high interannual stochasticity (as typical of Arctic regions; Kankaanpää et al. 2018), and what changes are actually due to long‐term, directional environmental change (Schmidt et al. 2023).

Overall, evidence of interaction strengths in the Arctic remains rare in the ecological literature. The shared cross‐boundary nature of the Arctic regions offers rich opportunities for intensive collaboration (Lemieux et al. 2025; Aronsson et al. 2021), also regarding this important element of biomonitoring. By identifying where interactions break down, we can anticipate failures and formulate adaptive responses. At the very least, we should capture the Arctic as it is now, building a solid baseline of ecological interactions for future studies. Creating multilayered ecological networks from standardized interaction data may offer a solution, as here attempted for the Svalbard archipelago.

## Author Contributions

**Mikhail K. Zhemchuzhnikov:** conceptualization (lead), formal analysis (lead), investigation (lead), methodology (lead), visualization (lead), writing – original draft (lead), writing – review and editing (equal). **Tomas Roslin:** conceptualization (lead), investigation (supporting), writing – original draft (supporting), writing – review and editing (equal). **Stephen J. Coulson:** conceptualization (supporting), investigation (supporting), writing – original draft (supporting), writing – review and editing (equal). **Joël Bêty:** conceptualization (supporting), investigation (supporting), writing – original draft (supporting), writing – review and editing (equal). **Anna Traveset:** conceptualization (lead), funding acquisition (lead), investigation (supporting), supervision (lead), writing – original draft (supporting), writing – review and editing (equal).

## Funding

The study is framed within project 101054177 “IslandLife”, funded by the European Research Council (ERC) and awarded to Anna Traveset.

## Conflicts of Interest

The authors declare no conflicts of interest.

## Supporting information


**Appendix S1:** Table of literature review outcomes.

**Appendix S2:** Table of scaled interaction strengths based on the literature review.

**Appendix S3:** Table of knowledge bias regarding ecosystem “flagship” nodes.

**Appendix S4:** Table of knowledge accumulation.

**Appendix S5:** Literature survey algorithm, supplementary tables, and figure.

**Appendix S6:** Python script (using SM2, SM3, and SM4).

## Data Availability

The data and code are available as Appendices S1–, S4 and S6.
