# Supplementary material for: Assessing the Ecological Network of Svalbard Through Scaled Interaction Strength Data: Insights From a Century of Research
Source: Ecol Evol. 2026 Mar 30;16(4):e73318. doi: 10.1002/ece3.73318 (PMC13107267; doi:10.1002/ece3.73318)
Supplement: Supplementary file 5 — Appendix S5: Literature survey algorithm, supplementary tables, and figure. [file ECE3-16-e73318-s003.pdf]

Appendix

Literature survey

The paper is focusing on both terrestrial and marine air-breathing organisms, bridging the two systems and examining how they are integrated across ecologically distinct environments within multiple layers of interactions.

The literature survey in Scopus was focused on the following Species/Functional groups of vertebrates and invertebrates:

Ecosystem node	Unit type	Taxon
Arctic fox	Terrestrial	Mammal
Svalbard reindeer	Terrestrial	Mammal
Barnacle goose	Terrestrial	Bird
Pink-footed goose	Terrestrial	Bird
Rock ptarmigan	Terrestrial	Bird
Shorebirds	Terrestrial	Bird
Snow bunting	Terrestrial	Bird
Red-throated diver	Terrestrial	Bird
Spiders	Terrestrial	Arthropod
Chironomid midges	Terrestrial	Arthropod
Dagger flies	Terrestrial	Arthropod
Parasitic wasps	Terrestrial	Arthropod
House flies	Terrestrial	Arthropod
Anthomyiid flies	Terrestrial	Arthropod
Sciarid midges	Terrestrial	Arthropod
Fungus gnats	Terrestrial	Arthropod
Dung flies	Terrestrial	Arthropod
Hoverflies	Terrestrial	Arthropod
Nematoceran larvae	Terrestrial	Arthropod
Brachyceran larvae	Terrestrial	Arthropod
Springtails	Terrestrial	Arthropod
Rove beetles	Terrestrial	Arthropod
Mites	Terrestrial	Arthropod
Blow flies	Terrestrial	Arthropod
Mosquitoes	Terrestrial	Arthropod
Aphids	Terrestrial	Arthropod
Polar bear	Marine	Mammal
Ringed seal	Marine	Mammal
Bearded seal	Marine	Mammal
Harp seal	Marine	Mammal
Walrus	Marine	Mammal
Eiders and ducks	Marine	Bird
Gulls	Marine	Bird
Skuas	Marine	Bird
Other seabirds	Marine	Bird
Greenland shark	Marine	Fish

To cover the full scope of available literature, containing the data on the interaction strength or newly observed interactions between organisms in Svalbard, we formulated our search inquiries for vertebrates and invertebrates as a combination of (1) an object of the study, (2) an interaction type and (3) location.

The inquiry for vertebrates

("Polar bear" OR "Ursus maritimus" OR "Arctic fox" OR "Vulpes lagopus" OR Reindeer OR caribou OR "Rangifer tarandus" OR seal* OR walrus OR phocid* OR Phoca OR Odobenus OR Pusa OR Cystophora OR Erignathus OR Pagophilus OR goose OR geese OR Anser OR Branta OR eider* OR Somateria OR duck* OR Clangula OR ptarmigan* OR "Lagopus muta" OR seabird* OR "marine bird" OR auk OR Alle OR Razorbill* OR Alca OR Cepphus OR Puffin OR Fratercula OR Kittiwake OR Rissa OR guillemot OR Uria OR shorebird* OR wader* OR Charadriiformes OR plover* OR Charadrius OR sandpiper* OR Calidris OR phalarope* OR Phalaropus OR dunlin* OR turnstone OR Arenaria OR gull* OR Larus OR Pagophila OR tern* OR Sterna OR skua* OR Stercorarius OR "Red-throated Diver" OR "Gavia stellata" OR "Northern Fulmar" OR "Fulmarus glacialis" OR "Northern Gannet" OR "Morus bassanus" OR "Snow Bunting" OR "Plectrophenax nivalis")

AND

(food OR diet OR foraging OR "feeding ecology" OR stomach OR scat OR prey OR faeces OR feces OR excrements OR "energy flow" OR "trophic transfer" OR "energy budget" OR "nutrient flow" OR "interaction strength")

AND

(Svalbard OR Spitsbergen OR "Barents Sea" OR "Kongsfjorden" OR Ny-Ålesund OR Longyearbyen OR Adventfjorden OR Isfjorden OR Hornsund OR Edgeøya OR Hopen OR Bjørnøya)

The inquiry for invertebrates

(arthropod* OR insect* OR invertebrat* OR mollusk* OR spider* OR Araneae OR Diptera OR Hemiptera OR Hymenoptera OR Coleoptera OR Acari OR Collembola OR Aphididae OR Anthomyiidae OR Calliphoridae OR Chironomidae OR Culicidae OR Empididae OR Heleomyzidae OR Muscidae OR Mycetophilidae OR Scathophagidae OR Sciaridae OR Sphaeroceridae OR Syrphidae OR Tenthredinidae OR Staphylinidae OR Curculionidae)

AND

(phytophag* OR Predat* OR Omnivor* OR granivor* OR herbivor* OR mycophag* OR scaveng* OR detrivor* OR decompos* OR pollinat* OR parasite* OR parasitoid* OR superparasite* OR symbiosis OR trophallaxis OR fluid-feed* OR cannib* OR kleptoparasite* OR phoresy OR blood* OR microbe* OR phytoplankton* OR interaction* OR diet OR food OR foraging OR "feeding ecology" OR stomach OR prey OR faeces OR feces OR excrements OR "energy flow" OR "trophic transfer" OR "energy budget" OR "nutrient flow" OR "interaction strength")

AND

(Svalbard OR Spitsbergen OR "Barents Sea" OR "Kongsfjorden" OR Ny-Ålesund OR Longyearbyen OR Adventfjorden OR Isfjorden OR Hornsund OR Edgeøya OR Hopen OR Bjørnøya)

On the 5th of May 2025, it resulted in a total of 704 and 239 papers for the inquiries on vertebrates and invertebrates. The papers were first selected by Titles and Abstracts, then by the content, which resulted in 71 papers, extra 4 papers were added when going through the initial 71, which at the end gave us 75 publications useful for our purpose to update the existing conceptual scheme of Svalbard archipelago Energy flow.

Supplementary tables and figures

Table S1. Knowledge bias across different groups of vertebrates studied on Svalbard archipelago. Knowledge: a total score showing how well the node was studied, we gave +1 for each species studied in a single paper, then we applied the importance correction factor (x 0.25) if that was a notable case study, but contained no data on interaction strength. Weighted knowledge: Knowledge to Species in nod ratio. E.g., node, which contains 5 species, is studied in 3 papers: the 1st paper contains the data on interaction strength for 1 species (score = 1 (1 x 1)), the 2nd paper contains the data on interaction strength for 2 species (score = 2 (2 x 1)), the 3rd paper is a case study for 1 species (score = 0.25 (1 x 0.25)). The sum of knowledge is 3.25. The weighted knowledge is corrected for the number of species in nod (5) = 3.25 : 5 = 0.65.

Node	Number of species	Knowledge	**Weighted knowledge**	Remark
Arctic fox	1	6.25	6.25	
Polar bear	1	6.25	6.25	
Glaucous gull	1	5.25	5.25	
Reindeer	1	4.25	4.25	
True seals	3	12	4	
Geese	3	8	2.67	regular breeders
Seabirds	14	36.5	2.61	regular breeders, excluding Glaucous gull
Rock ptarmigan	1	2	2	
Walrus	1	1.25	1.25	
Snow bunting	1	1	1	
Shorebirds	5	2	0.4	regular breeders

Table S2. Each interaction was assigned a score of 1, 2, 4, or 8 (with 0 indicating absence), following a log_2 progression to reflect the non-linear increase in relative interaction importance. These scores were based on the regularity of the interactions within and across years, and on its impact on consumers fitness and survival: No – no effect, “+” – observable impact, but not crucial on its own for energy-recipient, “+++” – strong impact on the fitness (and survival) of energy-recipient.

log_2(score)	Score	Regularity across years	Regularity within a year	Impact on consumer	Description	Examples
NA	**0**	No	No	No	**Absence** of interaction, or interaction is highly hypothetical **(0%)**	• Arctic fox hunting Ringed seal
0	**1**	No	No	No	**Marginal**, weak, occasional interaction (anecdotal cases), not regular across years or within years **(0 – 5%)**	• Polar bear hunting Reindeer<br>• Walrus hunting Barnacle goose<br>• Arctic fox scavenging on Seal carcass
1	**2**	Yes	No	"+"	**Auxiliary** interaction, regular across years, but sporadic in space and time within year, with limited impact on consumers fitness and survival, may be crucial when primary/keystone food source(s) is(are) not available **(5 – 20%)**	• Glaucous gull feeding on fish<br>• Ringed seal feeding on Crustaceans
2	**4**	Yes	Yes	"+"	Crucial **primary** interaction when the keystone resource is absent or scarce, or when the source is equally important with a few other resources **(20 – 67%)**	• Arctic fox scavenging on Reindeer and predating on seabirds and geese<br>• Shorebirds feeding on Diptera and Spiders
3	**8**	Yes	Yes	"+++"	**Keystone** interaction, critical for the survival of the energy recipient **(67 – 100%)**	• Polar bear hunting Ringed seals<br>• Little auks feeding on Crustaceans

Table S3. Scaled and averaged interaction strength. The values of interaction strength were used for building conceptual schematics of the ecological network. Although the initial scoring of interaction strength followed a discrete $\log_2$ progression (Table S2), averaging across studies results in intermediate values between the original scale categories. These averaged values were retained and rounded to one decimal place. Interaction types: Pr – Predation, Sc – Scavenging, He – Herbivory, Kl – Kleptoparasitism, Po – Pollination. Other abbreviations: m – migrate, mm – mostly migrate, f – frozen.

Consumer, common name	Consumer node	Resource	Interaction strength, summer	Interaction type	Remarks regarding summer profile	Interaction strength, winter	Interaction type	Remarks regarding winter profile
Arctic fox	Arctic fox	Cached food items	0.0	Pr		1.0	Pr	
Arctic fox	Arctic fox	Fish	0.6	Pr		2.0	Pr	
Arctic fox	Arctic fox	Geese	2.4	Pr		0.0	Pr	
Arctic fox	Arctic fox	Glaucous gull	1.2	Pr		2.0	Pr	extrapolation from IS with seabirds in winter
Arctic fox	Arctic fox	Ptarmigan	0.8	Pr		3.0	Pr	
Arctic fox	Arctic fox	Seabirds	4.0	Pr		2.0	Pr	
Arctic fox	Arctic fox	Shorebirds	0.4	Pr		0.0	Pr	
Arctic fox	Arctic fox	Snow bunting	0.4	Pr		0.0	Pr	
Arctic fox	Arctic fox	Svalbard reindeer	2.2	Sc		4.0	Sc	
Arctic fox	Arctic fox	True seals	0.4	Sc		2.5	Sc	
Arctic fox	Arctic fox	Walrus	0.0	Sc		1.0	Sc	

Arctic fox	Arctic fox	Polar bear (Svalbard reindeer)	0.1	Kl	case study: lowest score assigned	0.1	Kl	extrapolation from summer profile
Barnacle goose	Geese	Bryophyte	4.0	He		m	He	
Barnacle goose	Geese	Forbs	3.2	He		m	He	
Barnacle goose	Geese	Graminoids	3.2	He		m	He	
Barnacle goose	Geese	Prostrate shrubs	1.8	He		m	He	
Bearded seal	True seals	Crustaceans	4.0	Pr		4.0	Pr	based on summer dietary profile
Bearded seal	True seals	Fish	4.0	Pr		4.0	Pr	based on summer dietary profile
Bearded seal	True seals	Molluscs	4.0	Pr		4.0	Pr	based on summer dietary profile
Bearded seal	True seals	Polychaetes	0.0	Pr		0.0	Pr	based on summer dietary profile
Black guillemot	Other seabirds	Crustaceans	3.3	Pr		3.3	Pr	based on summer dietary profile
Black guillemot	Other seabirds	Fish	5.3	Pr		5.3	Pr	based on summer dietary profile
Black guillemot	Other seabirds	Molluscs	1.3	Pr		1.3	Pr	based on summer dietary profile
Black guillemot	Other seabirds	Polychaetes	0.0	Pr		0.0	Pr	based on summer dietary profile
Brent goose	Geese	Bryophyte	4.0	He		m	He	
Brent goose	Geese	Forbs	4.0	He		m	He	
Brent goose	Geese	Graminoids	2.0	He		m	He	
Brent goose	Geese	Prostrate shrubs	0.0	He		m	He	
Brunnich's guillemot	Other seabirds	Crustaceans	4.7	Pr		0, mm	Pr	
Brunnich's guillemot	Other seabirds	Fish	3.9	Pr		8, mm	Pr	
Brunnich's guillemot	Other seabirds	Molluscs	1.4	Pr		0, mm	Pr	
Brunnich's guillemot	Other seabirds	Polychaetes	0.7	Pr		0, mm	Pr	
Common guillemot	Other seabirds	Crustaceans	2.7	Pr		m	Pr	
Common guillemot	Other seabirds	Fish	5.7	Pr		m	Pr	
Common guillemot	Other seabirds	Molluscs	1.0	Pr		m	Pr	

Glaucous gull	Gulls and skuas	Crustaceans	1.0	Pr		0.0	Pr	
Glaucous gull	Gulls and skuas	Fish	2.0	Pr		8.0	Pr	
Glaucous gull	Gulls and skuas	Seabirds	8.0	Pr		0.0	Pr	
Glaucous gull	Gulls and skuas	Arctic fox (Seabirds)	0.1	Kl	case study: lowest score assigned	0.0	Kl	
Glaucous gull	Gulls and skuas	Common eider (Molluscs)	0.1	Kl	case study: lowest score assigned	0.0	Kl	
Glaucous gull	Gulls and skuas	Polar bear (Svalbard reindeer)	0.1	Kl	case study: lowest score assigned	0.1	Kl	based on summer dietary profile
Glaucous gull	Gulls and skuas	Geese	0.1	Pr	case study: lowest score assigned	0.0	Pr	
Greenland shark	Greenland shark	Crustaceans	0.5	Pr		0.5	Pr	based on summer dietary profile
Greenland shark	Greenland shark	Fish	8.0	Pr		8.0	Pr	based on summer dietary profile
Greenland shark	Greenland shark	Molluscs	1.0	Pr		1.0	Pr	based on summer dietary profile
Greenland shark	Greenland shark	Polychaetes	0.5	Pr		0.5	Pr	based on summer dietary profile
Greenland shark	Greenland shark	True seals	2.0	Pr		2.0	Pr	based on summer dietary profile
Grey phalarope	Shorebirds	Crustaceans	2.0	Pr		m	Pr	
Grey phalarope	Shorebirds	Diptera	4.0	Pr		m	Pr	
Grey phalarope	Shorebirds	Other invertebrates	2.0	Pr		m	Pr	
Grey phalarope	Shorebirds	Spiders	4.0	Pr		m	Pr	
Harbour seal	True seals	Crustaceans	1.0	Pr		m	Pr	
Harbour seal	True seals	Fish	8.0	Pr		m	Pr	
Harbour seal	True seals	Molluscs	1.0	Pr		m	Pr	
Harp seal	True seals	Crustaceans	6.0	Pr		m	Pr	
Harp seal	True seals	Fish	4.0	Pr		m	Pr	
Harp seal	True seals	Molluscs	0.3	Pr		m	Pr	
Kittiwake	Other seabirds	Crustaceans	3.0	Pr		0, mm	Pr	
Kittiwake	Other seabirds	Fish	6.5	Pr		8, mm	Pr	
Kittiwake	Other seabirds	Molluscs	0.0	Pr		0, mm	Pr	
Kittiwake	Other seabirds	Polychaetes	0.3	Pr		0, mm	Pr	
Kittiwake	Other seabirds	True seals	1.0	Pr		0, mm	Pr	

Little auk	Other seabirds	Crustaceans	8.0	Pr		m	Pr	
Little auk	Other seabirds	Fish	0.1	Pr		m	Pr	
Little auk	Other seabirds	Molluscs	0.1	Pr		m	Pr	
Little auk	Other seabirds	Polychaetes	0.1	Pr		m	Pr	
Northern fulmar	Other seabirds	Crustaceans	1.5	Pr		0.0	Pr	
Northern fulmar	Other seabirds	Fish	6.0	Pr		8.0	Pr	
Northern fulmar	Other seabirds	Molluscs	2.5	Pr		2.0	Pr	
Northern fulmar	Other seabirds	Polychaetes	2.5	Pr		0.0	Pr	
Pink-footed goose	Geese	Bryophyte	3.0	He		m	He	
Pink-footed goose	Geese	Forbs	4.0	He		m	He	
Pink-footed goose	Geese	Graminoids	4.0	He		m	He	
Pink-footed goose	Geese	Prostrate shrubs	1.0	He		m	He	
Polar bear	Polar bear	Geese	1.0	Pr		0.0	Pr	
Polar bear	Polar bear	Glaucous gull	1.0	Pr		1.0	Pr	
Polar bear	Polar bear	Seabirds	1.0	Pr		1.0	Pr	
Polar bear	Polar bear	Svalbard reindeer	0.0	Sc		0.7	Sc	
Polar bear	Polar bear	True seals	8.0	Pr		8.0	Pr	
Polar bear	Polar bear	Svalbard reindeer	0.1	Pr	case study: lowest score assigned	0.1	Pr	based on summer dietary profile
Ringed seal	True seals	Crustaceans	2.2	Pr		2.2	Pr	based on summer dietary profile
Ringed seal	True seals	Fish	6.4	Pr		6.4	Pr	based on summer dietary profile
Ringed seal	True seals	Molluscs	0.6	Pr		0.6	Pr	based on summer dietary profile
Ringed seal	True seals	Polychaetes	0.6	Pr		0.6	Pr	based on summer dietary profile
Rock ptarmigan	Ptarmigan	Bryophyte	1.8	He		4.0	He	suggested
Rock ptarmigan	Ptarmigan	Forbs	5.0	He		1.0	He	suggested
Rock ptarmigan	Ptarmigan	Graminoids	0.5	He		1.0	He	suggested
Rock ptarmigan	Ptarmigan	Prostrate shrubs	4.5	He		8.0	He	suggested
Snow bunting	Snow bunting	Diptera	8.0	Pr		m	Pr	
Snow bunting	Snow bunting	Other invertebrates	1.0	Pr		m	Pr	
Svalbard reindeer	Svalbard reindeer	Bryophyte	3.3	He		2.0	He	

Svalbard reindeer	Svalbard reindeer	Forbs	2.5	He		4.0	He	
Svalbard reindeer	Svalbard reindeer	Graminoids	4.5	He		4.0	He	
Svalbard reindeer	Svalbard reindeer	Prostrate shrubs	3.5	He		2.0	He	
Arctic tern	Other seabirds	Fish	8.0	Pr	extrapolation from overall profile	m	Pr	
Atlantic puffin	Other seabirds	Fish	8.0	Pr	extrapolation from overall profile	m	Pr	
Atlantic walrus	Atlantic walrus	Geese	1.0	Pr	extrapolation from overall profile	1.0	Pr	extrapolation from overall profile
Atlantic walrus	Atlantic walrus	Molluscs	8.0	Pr	extrapolation from overall profile	8.0	Pr	extrapolation from overall profile
Atlantic walrus	Atlantic walrus	True seals	1.0	Pr	extrapolation from overall profile	1.0	Pr	extrapolation from overall profile
Common eider	Other seabirds	Crustaceans	4.0	Pr	extrapolation from overall profile	m	Pr	
Common eider	Other seabirds	Molluscs	4.0	Pr	extrapolation from overall profile	m	Pr	
Common gull	Gulls and skuas	Crustaceans	4.0	Pr	extrapolation from overall profile	m	Pr	
Common gull	Gulls and skuas	Fish	4.0	Pr	extrapolation from overall profile	m	Pr	
Common gull	Gulls and skuas	Molluscs	4.0	Pr	extrapolation from overall profile	m	Pr	
Common gull	Gulls and skuas	Plants	4.0	Pr	extrapolation from overall profile	m	Pr	
European shag	Other seabirds	Fish	8.0	Pr	extrapolation from overall profile	m	Pr	
Great black-backed gull	Gulls and skuas	Fish	8.0	Pr	extrapolation from overall profile	m	Pr	
Great cormorant	Other seabirds	Crustaceans	2.0	Pr	extrapolation from overall profile	m	Pr	
Great cormorant	Other seabirds	Fish	8.0	Pr	extrapolation from overall profile	m	Pr	
Herring gull	Gulls and skuas	Crustaceans	4.0	Pr	extrapolation from overall profile	m	Pr	
Herring gull	Gulls and skuas	Fish	4.0	Pr	extrapolation from overall profile	m	Pr	
Ivory gull	Gulls and skuas	Crustaceans	2.0	Pr	extrapolation from overall profile	2.0	Pr	extrapolation from overall profile
Ivory gull	Gulls and skuas	Fish	8.0	Pr	extrapolation from overall profile	8.0	Pr	extrapolation from overall profile
Ivory gull	Gulls and skuas	Molluscs	0.0	Pr	extrapolation from overall profile	0.0	Pr	extrapolation from overall profile
Ivory gull	Gulls and skuas	Polychaetes	0.0	Pr	extrapolation from overall profile	0.0	Pr	extrapolation from overall profile
Northern gannet	Other seabirds	Fish	8.0	Pr	extrapolation from overall profile	m	Pr	
Razorbill	Other seabirds	Fish	8.0	Pr	extrapolation from overall profile	m	Pr	
Spiders	Spiders	Diptera	4.0	Pr	suggested based on knowledge from other Arctic sites	f	Pr	
Spiders	Spiders	Other arthropods	4.0	Pr	suggested based on knowledge from other Arctic sites	f	Pr	

Gulls and skuas	Gulls and skuas	Pinnipeds	0.1	Sc	suggested based on knowledge from other Arctic sites	0.1	Sc	suggested based on knowledge from other Arctic sites
Gulls and skuas	Gulls and skuas	Geese	0.1	Pr	suggested based on knowledge from other Arctic sites		Pr	
Gulls and skuas	Gulls and skuas	Svalbard reindeer	0.1	Sc	suggested based on knowledge from other Arctic sites	0.1	Sc	suggested based on knowledge from other Arctic sites
Prostrate shrubs	Diptera	Diptera	8.0	Po		f	Po	
Forbs	Diptera	Diptera	8.0	Po		f	Po	
Prostrate shrubs	Other arthropods	Other arthropods	2.0	Po		f	Po	
Forbs	Other arthropods	Other arthropods	2.0	Po		f	Po	

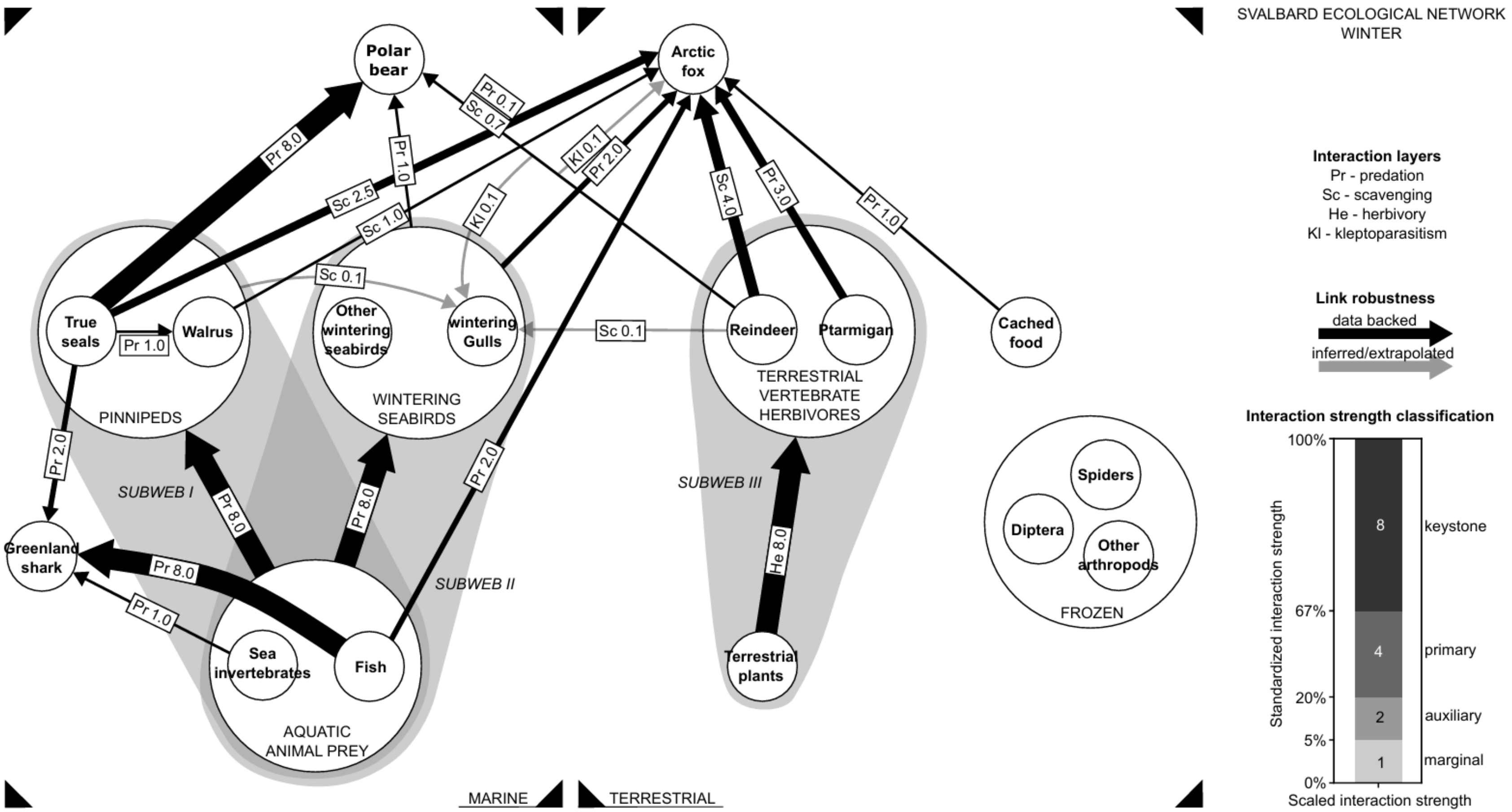

Figure S1. Proposed multilayered ecological network organisation. The figure shows a partial metaweb of interactions occurring in the **winter** in the Svalbard archipelago, with a focus on terrestrial and marine mammals and birds, and terrestrial invertebrates. Different interaction layers are indicated with prefixes: Pr – predation, Sc – scavenging, He – herbivory, Po – pollination, Kl – kleptoparasitism. The number after the prefix indicates scaled interaction strength, the arrow width is proportional to that value. Transparent arrows indicate understudied or extrapolated interactions, which are highly likely to occur but have never been the focus of exploring the Arctic ecological network in Svalbard.
